# Expression Dynamics of Core RNAi Machinery Genes in Pea Aphids Upon Exposure to Artificially Synthesized dsRNA and miRNAs

**DOI:** 10.3390/insects11020070

**Published:** 2020-01-21

**Authors:** Li Yang, Yuan Tian, Yuan-Yuan Peng, Jinzhi Niu, Jin-Jun Wang

**Affiliations:** 1 Key Laboratory of Entomology and Pest Control Engineering, College of Plant Protection, Southwest University, Chongqing 400716, China; y123456l@email.swu.edu.cn (L.Y.); t520321y@email.swu.edu.cn (Y.T.); grapeyuanyuan@163.com (Y.-Y.P.); jinzhiniu@swu.edu.cn (J.N.); 2 State Cultivation Base of Crop Stress Biology for Southern Mountainous Land, Academy of Agricultural Sciences, Southwest University, Chongqing 400716, China

**Keywords:** pea aphids, miRNA pathway, siRNA pathway, piRNA pathway

## Abstract

The pea aphid is an important pest of vegetables and causes serious losses worldwide. RNA interference (RNAi) is an effective pest control tool, and three sub-pathways have been described: The miRNA pathway, siRNA pathway, and piRNA pathway. A large number of genes in miRNA pathway and piRNA pathway are found to be expanded. To study the roles of these genes, the expression of 25 core RNAi genes was screened in spatiotemporal samples, artificially synthesized dsRNA and miRNA treated samples. The 25 genes were all expressed during different development stages and in different tissues. In dsRNA-treated samples and miRNA-treated samples, the expressions of genes in these three pathways were induced, especially the expanded genes. This suggests a complex network of RNAi core genes in the three sub-pathways. Treatment of miRNA seems to induce gene expression in a dosage-dependent manner. These results increase our knowledge of the siRNA pathway and related factors from RNAi pathway in aphids and promote the use of RNAi for the control of aphid pests.

## 1. Introduction

RNA interference (RNAi) involves post-transcriptional gene silencing. It silences complementary mRNA through a sequence-specific process [1]. RNAi occurs in all eukaryotes and affects biological activities, including developmental regulation, differentiation, and antiviral responses. The three RNAi pathways are microRNAs (miRNAs), small-interfering RNAs (siRNAs), and Piwi-interacting RNAs (piRNAs). They are classified depending on their region, biogenesis, mechanisms, biological roles, and functions [2]. The siRNA pathway, usually referred to as RNAi, is considered to have the most potential as a pest control strategy [3].

siRNAs are non-coding RNAs with 21–23 nts in length. Generally, siRNAs are produced from exogenous dsRNA (e.g., virus related dsRNA) while miRNA are endogenously transcribed. However, siRNA could also be produced from gonadal and somatic tissues in *Drosophila melanogaster* [4]. In the cytoplasm, dsRNA is cleaved into siRNA by Dicer2 [5], which is a highly conserved kind of dsRNA-specific endonuclease belonging to the RNase III family [6]. Subsequently, the siRNA interacts with the RNA-induced silencing complex (RISC) with the assistance of RISC-loading complex (RLC). The RLC was consisted of Argonaute2 and a dsRNA-binding partner protein, R2D2 which facilitate siRNA passage from Dicer2 to RISC [7]. The sense strand is sliced by Argonaute2, while the antisense strand remains in RISC [8]. Then, the RISC is guided to its target mRNA by the antisense strand by Argonaute2 which use siRNA recognizes and degrades the complementary mRNA in *Drosophila*. In comparison to miRNAs, siRNAs are fully complementary to target mRNA and specific genes. The miRNAs, a class of 19–24 nt single-stranded RNAs, are important in biological processes such as cell proliferation, apoptosis, and differentiation [9]. The miRNAs derive from endogenous genes encoding stem-loop primary RNAs (pri-miRNAs), which is typically transcribed from the nucleus into a primary product by RNA polymerase II [10]. In insects, the pri-miRNA is cleaved by the RNase III Drosha and the double-stranded RNA binding protein Pasha into pre-miRNAs (precursor microRNA) with a stem-loop structure of about 60–70 nts [11]. Drosha executes the initiation step of miRNA processing and the Pasha recognizes the substrate pri-miRNA [12]. With the assistance of Exportin-5 (Exp-5), pre-miRNAs are translocated to the cytoplasm. Then, mature miRNA (duplex RNA) is cleaved by another RNase III called Dicer1 and another double-stranded RNA binding protein called Loquacious which interact with Dicer1 and stimulates and directs the specific pre-miRNA processing activity [13]. miRNA is loaded into Ago1 which recognize the target gene to form RNA-induced silencing complex (RISC) where the Argonaute family is a key component. The RISC is guided to a target mRNA and inhibits target mRNA translation [14]. piRNAs are a class of non-coding small RNAs ranging from 24 to 30 nts in length. In the piRNA silencing pathway, Argonaute, Aubergine, and Piwi are the core components [15]. The piRNAs originate from a long single-stranded RNA which is transcribed from repetitive elements or genomic loci. In insects, the piRNAs are mainly derived from the genome of the transposable genetic element or a parasitic element of the host genome [16].

Expansion of portions of the miRNA and piRNA pathways has been found in aphids [17,18]. For siRNA pathway, one copy of *Dicer2*, *Ago2*, and *R2D2* were already identified in *D. melanogaster* [19]. However, two *Ago2* genes were reported in the *Culex pipiens* and *Musca domestica* [20,21] while three *Ago2* genes in *Glossina morsitans* [22]. Specially, *Tribolium castaneum* has two gene copies of *Ago2* and *R2D2* [23]. For miRNA pathway, both *Aedes aegypti* and *Diuraphis noxia* has two *Ago1* genes [21,24]. Besides, the *M. domestica* has two *Dicer1* genes [20]. For piRNA pathway, *G. morsitans* has three *Ago3* genes [25]. Two *Aubergine* genes which belong to the Piwi family were found in the *Nasonia vitripennis* and three in the *Harpegnathos saltator* [19]. However, 5–8 *Piwi* genes were reported in the *A. aegypti*, *Anopheles gambiae*, and *D. noxia* [21,24,25]. Duplications may lead to subfunctionalization or neofunctionalization in RNAi pathways and could explain different RNAi efficacy in insects. For Ago proteins, Agos belong to a multigene family that is divided into Agonaute and Piwi subgroups [26]. Different types of Ago-type proteins act in different steps of the exo-siRNA or endosiRNA pathway. A study on *A. aegypti* showed that the expansion of the Piwi protein family might allow Piwi proteins to be functionally specialized in the biogenesis of piRNAs from different origins [27]. The insect species studied so far have only one Pasha, but four Pasha-like genes have been found in the pea aphid, *Acyrthosiphon pisum*. The difference among the four Pasha proteins in the pea aphid involves a block of 95 AA at the N-terminal end. This block is repeated three times and two times in Pasha1 and Pasha2 while is absent from Pasha3 and Pasha4. However, the sequence of this 95 AA has no homology in the Interpro database and its functional significance is unknown [17]

In *D. melanogaster* S2 cells, RNAi-mediated downregulation of the *GFP* reporter included the expression of genes in siRNA, *Ago2,* and *Dicer2*. In contrast, silencing *Dicer1* and *Ago1* inhibited *GFP* silencing [28]. In *Manduca sexta*, *Dicer2* and *Ago2* mRNA levels were elevated following injection with dsRNA in a specific and dose dependent manner [29]. In *A. gambiae*, some of the piRNA pathway genes are involved in the exogenous dsRNA-induced silencing response [30]. Besides, the mosquitos’ soma which infected with transgenic CHIKV expressing the dsRNA-binding protein B2 suggests that dsRNA molecules are an inducer of the piRNA pathway [31].

*Aphis pisum* (Hemiptera: Aphididae) is a significant vegetable pest that causes large economic losses. In *A. pisum*, one copy of *Dicer2*, *Ago2*, and *R2D2* was found in the siRNA pathway [32]. One copy of *Drosha*, *Exp-5*, duplications of *Ago1* (*Ago1a* and *Ago1b*), *Dicer1* (*Dicer1a* and *Dicer1b*), and *Loquacious* (*Loquacious1* and *Loquacious2*), four copies of *Pasha* (*Pasha1*, *Pasha2*, *Pasha3,* and *Pasha4*) in the miRNA pathway were reported [17]. As for piRNA pathway, Piwi proteins are divided into Piwi and Aub. Duplication of *Ago3* (*Ago3a* and *Ago3b*) and eight copies of *Piwi/Aub* (*Piwi1*, *Piwi2*, *Piwi3*, *Piwi4*, *Piwi5*, *Piwi6*, *Piwi7*, and *Piwi8*) of the piRNA pathway have also been reported [18]. The Aubergine belongs to the Piwi protein in pea aphid by BLAST the sequences of *D. melanogaster* and the study [17]. So we kept its name *Piwi.* Currently, researches on these genes have focused on bioinformatics. Identification, evolutionary rate, and phylogenetic analysis were studied by bioinformatics approach [17,18]. However, these genes are rarely investigated using experimental approach. There is a need to study three pathways of RNAi activity as a possible aphid control strategy [32,33,34]. To explore the potential role of these expanded genes in three sub-pathways of RNAi, the expressions of 25 core RNAi genes were detected in different development stages, various tissues, dsRNA-treated samples, and miRNA-treated samples. The hypothesis was that there may be crosstalk among miRNA pathway, siRNA pathway and piRNA pathway. These results show a complex network of RNAi core genes in the three sub-pathways.

## 2. Materials and Methods

### 2.1. Pea Aphid Strain

The genome strain of pea aphid was originally provided, in 2016, by Professor Guy Smagghe (Ghent University, Belgium). The strain was reared on broad bean (*Vicia faba*) at 25 ± 1 °C, 75 ± 5% relative humidity, and a 14:10 (L:D) photoperiod.

### 2.2. Sample Preparation for Different Development Stages and Tissues

For studies on the different development stages, 500 pea aphid adults were initially cultured on broad bean seedlings, and we collected samples from succeeding generations. The samples of N^1st^-1, N^1st^-2, and N^1st^-3 were collected in the early 4 h, middle time, and the last 4 h before the next molt of the 1st instar nymph, respectively. Samples from other nymph stages were taken using the same pattern. Additionally, the Ad-1, Ad-2, Ad-3, and Ad-4 were collected from 1, 9, 12, and 15 d adults. Several tissues including epidermis, embryo, gut, fat body, brain, stylet, muscle, and hemolymph were isolated from adults within 12 h after molting. Four biological replicates were performed for each sample. The samples were frozen in liquid nitrogen and kept at −80 °C for future use.

### 2.3. RNA Extraction

TRIzol reagent (Invitrogen, Carlsbad, CA, USA) was used to extract total RNA using manufacturer’s instructions. The concentration and the purity of total RNA were evaluated at OD260/280 and OD260/230 using a NanoDrop One (Thermo Scientific, Wilmington, DE, USA). RNA integrity was confirmed by 1% agarose gel electrophoresis.

### 2.4. First-Strand cDNA Synthesis for mRNA

One μg total RNA was treated with RQ1 RNase-Free DNaseI (Promega, Madison, WI, USA) to remove the genomic DNA. A PrimerScript RT Reagent Kit (Takara, Dalian, China) was used to synthesize the first strand cDNA. Then, the synthesized cDNA was stored at −20 °C until use.

### 2.5. dsRNA Synthesis and Delivery

The primers used for the dsRNA synthesis were designed using Primer 3.0 (http://bioinfo.ut.ee/primer3-0.4.0/) and are listed in Table S1. According to our previous study, *Hunchback* (*HB*) was used as the indicator gene to calculate gene silencing efficiency. This gene is functionally involved in abdominal identity suppression and germband growth [35]. DsRNA of *HB* and *Green fluorescent protein* (ds*GFP*) were synthesized in vitro using a Transcript Aid T7 High Yield Transcription Kit (Thermo Scientific, Wilmington, DE, USA) basing on manufacturer protocol. The final concentration of ds*HB* (ds*GFP*) was diluted into 60 ng/μL, 600 ng/μL, and 6000 ng/μL by nuclease-free water. 10 nL has 0.6 ng, 6 ng, and 60 ng for 60 ng/μL, 600 ng/μL, and 6000 ng/μL was injected into dorsal part of adults’ abdomens using a M3301 micromanipulator (World Precision Instruments, Sarasota, FL, United States). After injection, aphids were put on broad bean seedlings and 36 h, the aphids were collected for RNA extraction. We choose 36 h to collect samples based on our previous study [36]. The RNAi efficiency of the targeted gene was optimal at this time point. Four biological replicates were performed for each treatment.

### 2.6. microRNA Agomir/Antagomir Synthesis and Delivery

An aphid specific expressed miRNA, *miR-3024*, was used as a miRNA indicator. The function of *miR-3024* is still unknown while it was indicated to be involved in aphid-Buchera interactions [37]. The *miR-3024* agomir/antagomir and agomir/antagomir negative controls were synthesized by Ribobio (Guangzhou, China). A 10 nL amount of each agomir/antagomir was injected into adults. The final dosages of *miR-3024* agomir/antagomir were 1 pmol (agomir 15.4 ng; antgomir 8 ng), 1.5 pmol (agomir 23.1 ng; antgomir 12 ng) and 2 pmol (agomir 30.8 ng; antgomir 16 ng). After injection, aphids were put on broad bean seedlings and 36 h later, the aphids were collected for RNA extraction. Four biological replicates were performed in each treatment.

### 2.7. Quantitative Real-Time Polymerase Chain Reaction (RT-qPCR) for mRNA

The primers for quantitative reverse transcription PCR (RT-qPCR) were designed using Primer 3.0 (http://bioinfo.ut.ee/primer3-0.4.0/) and listed in Table S1. All primers were evaluated using a serial dilution of one cDNA sample to assure amplification efficiency and specificity. RT-qPCR was performed on a BIO-RAD CFX Connect Real-Time System (Bio-Rad, Hercules, CA) using the NovoStar SYBR RT-qPCR SuperMix (Novoprotein Scientific, Shanghai, China). The reactions were performed in a 10 μL volume of a mixture containing 5 μL SYBR Green mix, 0.5 μL cDNA template, 0.5 μL of each primer and 3.5 μL nuclease-free water. The amplication program was a two-step method and its program was as follows: 95 °C for 2 min, followed by 40 cycles of 95 °C for 1.5 s, 60 °C for 30 s. A melt curve analysis from 60 °C to 95 °C was conducted to ensure specificity. The *elongation factor-1 alpha* (*EF1α*) and *ribosomal protein S2-like* (*RPS2*) were used as reference genes [38,39]. Four biological replicates were performed for each sample and the relative expression levels were analyzed using qBase software based on the 2^ΔΔ-CT^ method [40].

### 2.8. First Strand cDNA Synthesis of miRNA

Two μg total RNA was purified using phenol chloroform (phenol water: chloroform (*v*/*v*) = 1:1). Before the first-strand cDNA of miRNA was synthesized, the miRNA was added to the poly(A) using a miRNA cDNA synthesis kit (Tiangen, Beijing, China) based on the manufacturer’s instructions. The poly(A) reaction contained 2 μg purified RNA, 2.5 μL 1×poly(A) polymerase buffer, 2.5 μL ATP, 0.5 μL *E. coli* poly(A) polymerase and RNase-free water up to 25 μL. The reaction condition was 37 °C for 15 min. Then, the first cDNA of miRNA was synthesized in a 20 μL volume of a mixture containing 1 μL ultrapure dNTP mix, 3 μL RT primers, 4 μL 5×superRT buffer, 0.5 μL superRT, 7.5 μL RNase-free water and 4 μL poly(A) reaction fluid. The reaction program was 42 °C for 50 min and 85 °C for 5 min. The cDNA was stored at −20 °C until use.

### 2.9. RT-qPCR for miRNA

RT-qPCR was performed on a BIO-RAD CFX Connect Real-Time System using a miRNA RT-qPCR assay kit (Tiangen, Beijing, China). The mature *miR-3024* gene sequence was used as the forward primer and the U6 snRNA was used as a reference [41] (Table S1). The reactions were performed in a 20 μL volume of a mixture containing 1 μL 2×miRNA RT-qPCR mix, 1 μL cDNA template, 0.4 μL of each primer, and 8.2 μL of nuclease-free water. The program was as follows: 95 °C for 1 min, followed by 40 cycles of 95 °C for 1.5 s, 60 °C for 1 min, and 60 °C for 1 min. A melt curve analysis from 60 °C to 95 °C was conducted to ensure specificity for all reactions. Four biological replicates were performed for each sample. The relative expression levels were analyzed using qBase software based on the 2^ΔΔ-CT^ method.

### 2.10. Statistical Analysis

All statistical analyses were performed with SPSS 16.0 for Windows (SPSS Inc., Chicago, IL, USA). Significant differences in transcript levels of different issues, development stages, dsRNA and miRNA agomir/antagomir were tested by one-way analysis of variance (ANOVA), followed by Tukey’s honestly significant difference multiple comparison test (*p* < 0.01). The Significant differences of dsRNA and miRNA agomir/antagomir was analyzed in each dosage, independently.

## 3. Results

### 3.1. Spatiotemporal Expression Profiles of Core Genes of Three RNAi Pathways in Different Developmental Stages and Different Tissues of the Pea Aphid

**Figure 1:**
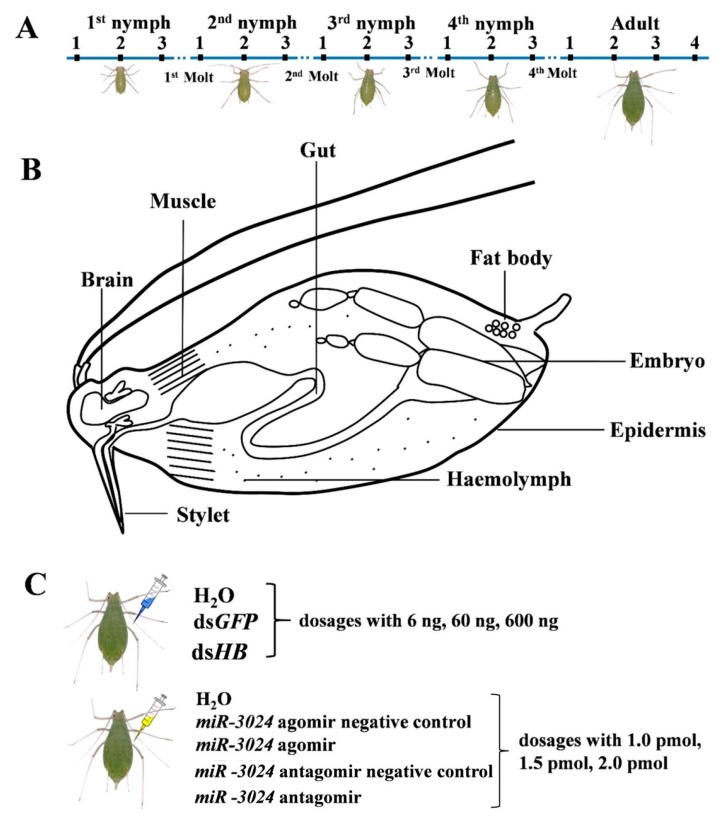
Experimental design. (**A**) Sample collection from different developmental stages. N^1st^-1: new-born nymphs at 4 h; N^1st^-2: middle of first instar nymphs; N^1st^-3: first instar nymphs 4 h before the first molt; N^2nd^-1: second instar nymphs 4 h after the first molt; N^2nd^-2: middle of second instar nymphs; N^2nd^-3: second instar nymphs 4 h before the second molt; N^3rd^-1: third instar nymphs 4 h after the second molt; N^3rd^-2: middle of third instar nymphs; N^3rd^-3: third instar nymphs 4 h before the third molt; N^4th^-1: fourth instar nymphs 4 h after the third molt; N^4th^-2: middle of fourth instar nymphs; N^4th^-3: fourth instar nymphs 4 h before the fourth molt; Ad-1: adult 4 h after the fourth molt; Ad-2: aphids at 9 d; Ad-3: aphids at 12 d; Ad-4: aphids at 15 d. (**B**) Sample collection of different tissues. Eight tissues (gut, epidermis, muscle, brain, fat body, embryo, stylet, and hemolymph) of pea aphids were dissected and collected. (**C**) Sample collections from different dosages of dsRNA treatments and miRNA agomir/antagomir treatments. For microinjection, each pea aphid was injected with 10 nL ds*HB* or *miR-3024* agomir/antagomir. ds*GFP* and *miR-3024* agomir negative control/antagomir negative control as control. 1 pmol: 15.4 ng (agomir), 8 ng (antagomir); 1.5 pmol: 23.1 ng (agomir), 12 ng (antagomir); 2 pmol: 30.8 ng (agomir), 16 ng (antagomir).

**Figure 2:**
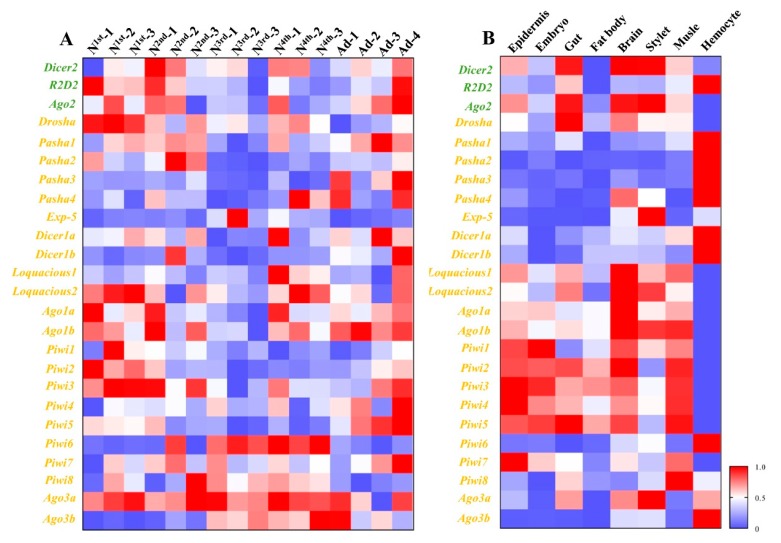
Spatiotemporal expression profiles of core genes of three RNA interference (RNAi) pathways in different developmental stages and different tissues. (**A**) Expression profiles of core genes of three RNAi pathways among different developmental stages. (**B**) Expression profiles of core genes of three RNAi pathways among different tissues. Mean (±SE) expression level was based on four biological replicates. The relative expression was normalized based on two reference genes, *A. pisum elongation factor-1 alpha* (*EF1a*) and *ribosomal protein S2-like* (*RPS2*). All values are homogenized using the minimum maximum method. The green letters, orange letters, and purple letters respectively represent the genes in siRNA pathway, miRNA pathway, and piRNA pathway.

Different developmental stages and different tissues of pea aphid adults were collected (Figure 1), and the transcriptions of 25 genes from three RNAi pathways, miRNA, siRNA, and piRNA were measured by RT-qPCR (Figure 2). The results showed most genes were all expressed in all developmental stages and different tissues. *Exp-5* only exhibited high expression in the middle of third instar nymphs, and *Piwi6* showed high expression in third and fourth instar nymphs. The transcript levels of *Ago1a*, *Ago1b*, *Dicer1a*, *Dicer1b*, *Pasha1*, *Pasha2*, *Pasha3*, *Pasha4*, *Ago2*, *R2D2*, *Piwi1*, *Piwi2*, *Piwi5*, and *Piwi7* were lower in the old nymphs, but higher in the young nymphs and adults. However, *Exp-5*, *Ago3b*, *Piwi6*, and *Piwi8* possessed contrasting expression patterns. The expression levels of the remaining genes were not different among developmental stages (Figure 2). The relative expressions of most genes were below 1.2 while *Pasha3*, *Pasha4*, *Exp-5*, *Piwi4*, *Piwi6*, and *Ago3b* were greater than 1.2 in fourth instar nymphs and adults. Expression of *Exp-5* and *Piwi6* exceeded 3.

Epidermis, embryo, gut, fat body, brain, stylet, muscle, and hemolymph were dissected from adults for expression pattern analyses (Figure 2). The greatest expression change of *Pasha4* was observed in the hemolymph. *Exp-5*, *Ago2*, *Ago3b*, and *Piwi6* were highly expressed in the brain, stylet and hemolymph. *Ago1a*, *Ago1b*, *Dicer1b*, *Drosha*, *Piwi3*, *Piwi4*, *Piwi5*, and *Piwi7* had high expression in seven tissues, except for the hemolymph. In contrast, *Dicer1b*, *Pasha1*, *Pasha2*, *Pasha3*, and *R2D2* had high expression levels in hemolymph but not in the other tissues. The expression levels of *Dicer1a* and *Dicer2* were similar among the sampled tissues (Figure 2).

### 3.2. Expression Profiles of Core Genes of Three RNAi Pathways Upon dsRNA Treatment

**Figure 3:**
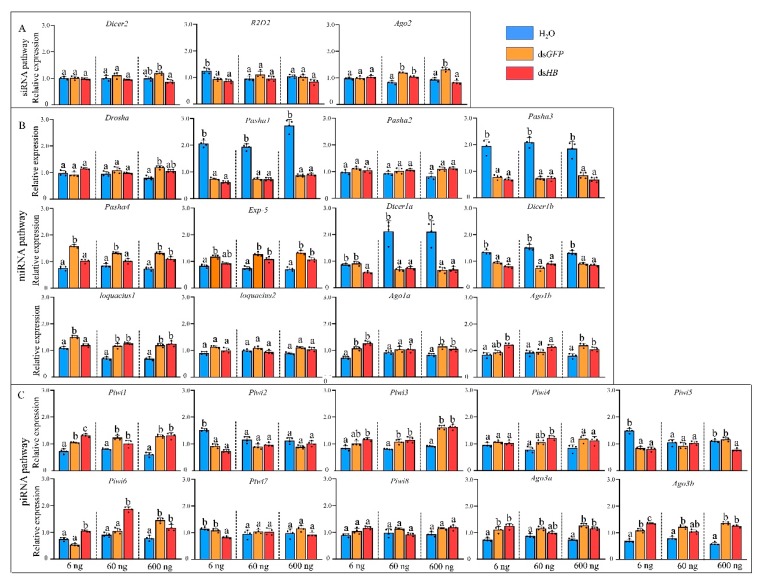
Expression profiles of core genes of three RNAi pathways upon dsRNA administration by microinjection. (**A**) siRNA pathway. (**B**) miRNA pathway. (**C**) piRNA pathway. Different colors represent different level of changes. Mean (±SE) expression level was based on four biological replicates. The relative expression was normalized based on two reference genes, *A. pisum elongation factor-1 alpha* (*EF1α*) and *ribosomal protein S2-like* (*RPS2*). Lowercase letters above each bar indicate significant differences (one-way ANOVA followed by Tukey’s honestly significant difference multiple comparison test; *p* < 0.01). The Significant difference was analyzed in each dosage, independently.

To study the responses of three RNAi pathways (siRNA, miRNA, and piRNA) after dsRNA treatment (Figure 1), the expressions of 25 genes of the core machinery of RNA pathways were examined after injection of different dsRNA doses. After 6 ng ds*HB* treatment, a gene, *R2D2*, in the siRNA pathway, was downregulated. Two miRNA genes (*Ago1a* and *Ago1b*) and five piRNA pathway genes (*Piwi1*, *Piwi3*, *Piwi6*, *Ago3a*, and *Ago3b*) were significantly upregulated. Surprisingly, four miRNA pathway genes (*Pasha1*, *Pasha3*, *Dicer1a*, and *Dicer1b*) and two piRNA pathway genes (*Piwi2*, *Piwi5*, and *Piwi7*) were significantly downregulated (Figure 3). After 60 ng ds*HB* treatment, *Ago2* was upregulated and the expression levels of miRNA and piRNA pathway genes were altered. Among these, *Exp-5*, *Loquacious1*, *Piwi1*, *Piwi3*, *Piwi4*, and *Piwi6* were significantly upregulated, while *Pasha1*, *Pasha3*, *Dicer1a*, and *Dicer1b* were significantly downregulated (Figure 3). Upon 600 ng ds*HB* treatment, the expression of *HB*, was significantly reduced (Figure S1). Within the same treatment, expressions of siRNA pathway genes were not significantly different. However, five miRNA pathway genes (*Pasha4*, *Exp-5*, *Loquacious1*, *Ago1a*, *Ago1b*) and five piRNA pathway genes (*Piwi1*, *Piwi3*, *Piwi6*, *Ago3a*, *Ago3b*) were significantly upregulated whereas four miRNA pathway genes (*Pasha1*, *Pasha3*, *Dicer1a*, *Dicer1b*) and one piRNA pathway gene (*Piwi5*) were significantly downregulated (Figure 3). Compared to the ds*HB*, three miRNA pathway genes (*Pasha4*, *Dicer1a*, and *Loquacious1*) and one piRNA pathway gene (*Piwi7*) were significantly downregulated while one piRNA pathway gene (*Piwi6*) was upregulated in 6ng. One miRNA pathway gene (*Pasha4*) was downregulated while one piRNA pathway gene (*Piwi6*) was upregulated in 60 ng. As for 600 ng, two siRNA pathway genes (*Dicer2*, *Ago2*) and one piRNA gene (*Piwi5*) were downregulated.

### 3.3. Expression Profiles of Core Genes of Three RNAi Pathways Upon miRNA Treatment

**Figure 4:**
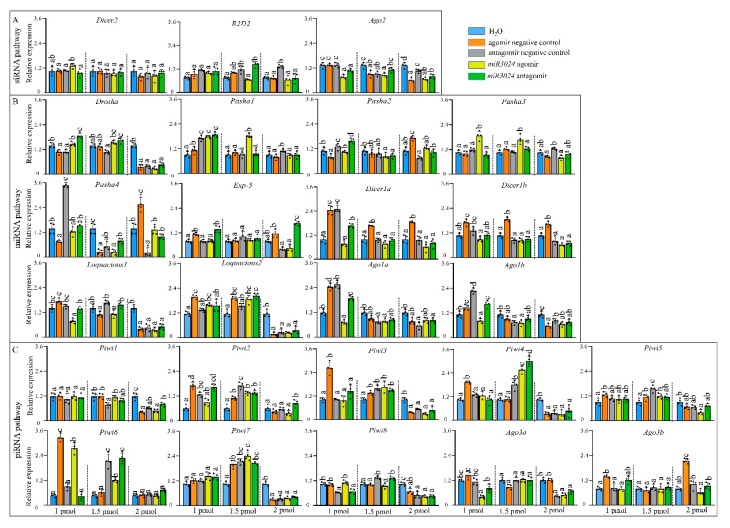
Expression profiles of core genes of three RNAi pathways after miRNA agomir/antagomir administration by microinjection. (**A**) siRNA pathway. (**B**) miRNA pathway. (**C**) piRNA pathway. Different colors represent different change levels. Mean (±SE) expression level is based on four biological replicates. The relative expression was normalized based on two reference genes, *A. pisum elongation factor-1 alpha* (*EF1a*) and *ribosomal protein S2-like RPS2*. Lowercase letters above each bar indicate significant differences (one-way ANOVA followed by Tukey’s honestly significant difference multiple comparison test; *p* < 0.01). agoNC: agomir negative control; antNC: antagomir negative control; ago3024: *mi-R3024* agomir; ant3024: *miR-3024* antagomir. The Significant difference was analyzed in each dosage, independently. 1 pmol: 15.4 ng (agomir), 8 ng (antagomir); 1.5 pmol: 23.1 ng (agomir), 12 ng (antagomir); 2 pmol: 30.8 ng (agomir), 16 ng (antagomir).

Expression levels of the 25 genes were detected after the treatment with different dosages of *miR-3024* agomir/antagomir (Figure 4). The altered expression level of *miR-3024* was also confirmed to verify the success of experimental treatments (Figure S2). After 1 pmol agomir treatment, the expression of *Ago2* was downregulated while three miRNA pathway genes (*Pasha1*, *Pasha3*, *Loquacious2*) were significantly upregulated and three miRNA pathway genes (*Loquacious1*, *Ago1a*, *Ago1b*) were significantly downregulated. One piRNA pathway genes (*Ago3a*) were significantly downregulated. Upon 1.5 pmol agomir treatment, the expressions of siRNA pathway genes *Ago2* decreased. Three miRNA pathway genes (*Pasha1*, *Pasha3*, *Loquaciou2*) were upregulated and four genes (*Pasha2*, *Pasha4*, *Ago1a*, and *Ago1b*) were downregulated while five piRNA pathway genes (*Piwi2*, *Piwi4*, *Piwi5*, *Piwi6*, and *Piwi7*) were upregulated. In the 2 pmol agomir treatment, the expression of *Ago2* in the siRNA pathway was downregulated (Figure 4). Four miRNA pathway genes (*Drosha*, *loquacious1*, *loquacious2*, and *Ago1b*) were downregulated. In the piRNA pathway, *Piwi1*, *Piwi3*, *Piwi4*, *Piwi5*, *Piwi7*, *Piwi8*, and *Ago3a* were downregulated. In terms of dosage, the expressions of 9 genes (*Ago2*, *Pasha1*, *Pasha3*, *Loquacious1*, *Loquacious2*, *Ago1a*, *Ago1b*, *Piwi6*, and *Ago3a*) were changed in 1 pmol while 13 genes (*Ago2*, *Pasha1*, *Pasha2*, *Pasha3*, *Pasha4*, *Loquacious2*, *Ago1a*, *Ago1b*, *Piwi2*, *Piwi4*, *Piwi5*, *Piwi6*, and *Piwi7*) in 1.5 pmol and 12 genes (*Ago2*, *Pasha1*, *Loquacious*, *Loquacious2*, *Ago1a*, *Ago1b*, *Piwi1*, *Piwi3*, *Piwi4*, *Piwi5*, *Piwi7*, *Piwi8*, and *Ago3a*) in 2 pmol.

## 4. Discussion

With the expansion of core RNAi genes discovered in pea aphids, it remains unclear how these contribute to the function of RNAi pathways. To explore the function of these genes the transcript levels of a number of 25 core genes in three RNAi pathways (siRNA, miRNA, and piRNA) were screened in three experimental conditions: spatiotemporal samples, artificial synthesized dsRNA treated samples, and artificial synthesized miRNA treated samples.

In the spatiotemporal samples, we determined the transcription level of the 25 genes in developmental stages and tissues. The expression dynamics of these genes were variable. Genes with the presented expression based on the RT-qPCR detection limit, and the variation expression levels in certain developmental stages or tissues provide clues to their involvement in specific physiological processes. *Pasha3*, *Pasha4*, *Exp-5*, *Piwi4*, *Piwi6*, and *Ago3b* had high expression in fourth instar nymphs and adults, which are critical stages in maturation and reproduction, respectively. These genes are closely associated with miRNA and piRNA, and these two pathways are important in the regulation of development and reproduction [42,43]. piRNA pathway is known to function primarily in suppressing transposon expression in the gonads of animals and *Ago3* is expressed specifically in germ cells of *D. melanogaster* [44]. However, the pea aphid used in our study is parthenogenesis. The expression of *Ago3* is still unknown in tissues of aphids of sexual reproduction phase.

**Figure 5:**
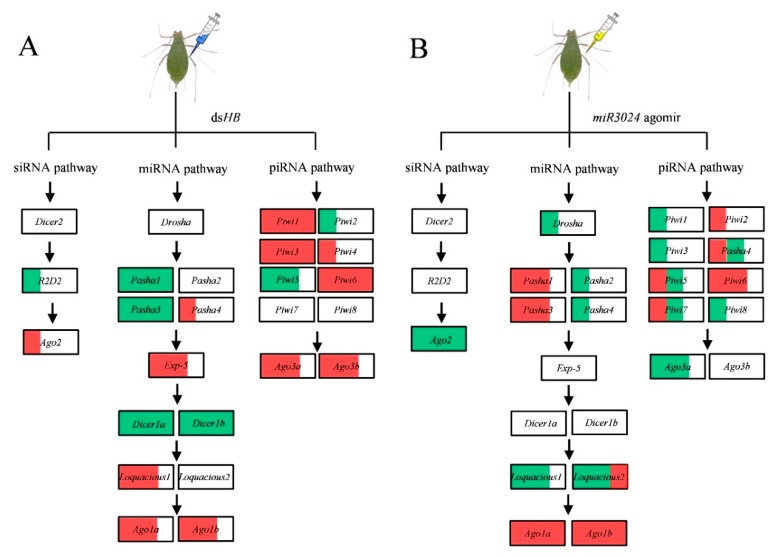
Schematic of expression profiles of core genes of three RNAi pathways upon dsRNA and miRNA agomir administration by microinjection. (**A**) ds*HB* treatment. (**B**) *miR-3024* agomir treatment. The white, red, and green of the box represents the expression of gene are no significance, upregulation, and downregulation, respectively. 1/3, 2/3, and full red/green area of the box represent the ratio of genes upregulation/downregulation in three dose treatments of dsHB (6 ng, 60 ng, 600 ng) or *miR-3024* agomir/antagomir (agomir: 15.4 ng, 23.1 ng, 30.8 ng; antagomir: 8 ng, 12 ng, 16 ng).

RNAi (normally inferred to as the siRNA pathway) is a promising tool for aphid control [32,33,34] but RNAi use in effective pest control remains a challenge. This is due to gene silencing efficacies (both RNA and protein level), pest control efficacy (mortality), field delivery systems and potential resistance development [44]. Therefore, we evaluated the transcriptional level of 25 genes in artificial synthesized dsRNA treated samples. As expected, genes of the siRNA pathway (e.g., *Ago2*) were upregulated after dsRNA treatment showing that the exogenous application of dsRNA induced the activity of the RNAi pathway [45]. However, two other genes, *Dicer2* and *R2D2*, were not significantly altered after the same dsRNA treatment. Of the three dsRNA doses (6, 60, 600 ng per aphid), only the 600 ng treatment induced significant gene silencing of the target gene (*HB*). The induced level of *Ago2* only had a slight difference. This trend occurred in the ds*GFP* treated sample but not in the ds*HB* treated sample. We previously observed that the induction of these three genes occurred 24 h after dsRNA injection (600 ng per aphid) [36], implying that the variation of gene expression level induction is associated with the time of sample collection and the physiological stage of the aphids. We believe the expressions of 25 core genes upon the dsRNA treatment are dose-dependent as well as in sufficient dose presenting a time points-based dynamics. For instance, the response of core machinery was detected in both 24 h [36] and 36 h in the current study. In addition, the RNAi efficiency of the targeted gene was the highest at the 36 h [36]. Under the same treatment, there were significant expression changes in miRNA and piRNA pathway associated genes. For miRNA, reduction of *Pasha1*, *Pasha3*, *Dicer1a*, and *Dicer1b* were observed in three dsRNA dosages (6, 60, 600 ng), while induction of *loquacious 1*, *Ago1a*, and *Ago1b*, were observed upon dsRNA treatments, reflecting a plasticity of miRNA pathways upon dsRNA in pea aphids. For piRNA, the induction of *Piwi1*, *Piwi3*, *Piwi6*, *Ago3a*, and *Ago3b* occurred after dsRNA treatments (Figure 5). The genes in siRNA pathway would respond to the dsRNA [28]. But ds*HB* target its mRNA while ds*GFP* act as a non-target negative control, this may differ the response of the siRNA pathway core genes between treatments of ds*GFP* and ds*HB*. For example, *Ago2* upregulated upon 600 ng of ds*GFP* while it did not significantly alter upon ds*HB*. A crosstalk existed among miRNA pathway, siRNA pathway and piRNA pathway [28,29]. Thus, the difference of ds*GFP* and ds*HB* treatments could also affect the response of genes in miRNA pathway (e.g., *Pasha4*) and piRNA pathway (e.g., *Piwi6*). siRNAs and miRNAs are Dicer-dependent groups and piRNAs are the Dicer-independent Group. Competition with the Argonaute protein could be associated with above-observed phenomenon [46]. In addition, genes such as *Loquacious* could function as interactive genes to connect the miRNA pathway and the siRNA pathway [35]. Thus, the pea aphid genes altered after dsRNA treatments, which are normally recognized as genes in the miRNA/piRNA pathway, should be studied to determine if they contribute to the siRNA pathway activity. This could enhance our understanding of the siRNA pathway mechanism in aphids and its associated factors. These data will be needed to design an efficient RNAi gene silencing approach for aphid pest control. The study in *Drosophila* showed that *Ago2* and *Dicer2* downregulated in 2 ug of ds*GFP* treatment [28] But in pea aphids *Ago2* were upregulated in dsRNA treatments of 60 ng and 600 ng rather than 6 ng. It seems that the change of genes in RNAi pathways may be relative to dose of dsRNA. Dose-dependent upregulation of *Dicer2* and *Ago2* expression was also found in response to injection with dsRNA in *M. sexta* [29]. Exogenous dsRNA induced the gene expressions of the piRNA pathway were also reported [30,31] which was also observed in *A. pisum* in this study. Taken together, it can indicate crosstalk among miRNA pathway, siRNA pathway, and piRNA pathway.

We used the same experimental approach, with artificially synthesized miRNA agomir and antagomir (e.g., *miR-3024*), to detect the responses of these 25 genes. The gene expression responses to dsRNA treatment were complex. It seems that the treatment of artificially synthesized miRNA induced dosage dependent gene expression. At the low dose (1 pmol), gene expressions such as *Pasha1*, *Pasha3*, *Ago1a*, *Ago1b*, and *Piwi6* were induced. With the 1.5 pmol dose, expression of genes such as *Piwi4* and *Piwi7* were induced. In the 2 pmol dose, genes such as *Drosha*, *Loquacious1*, *Loquacious2*, *Piwi4*, *Piwi7*, and *Ago3a* were down regulated. These data indicate that miRNA and piRNA-associated genes can interact with these expanded genes in a way similar to the siRNA pathway. This suggests a complex network of RNAi core genes in the three sub-pathways. siRNAs injected into the embryos of zebrafish competed for cofactors that were required in the miRNA pathway [46]. Similarly, in mammalian cells, transfection of siRNAs can alter the levels of many miRNAs by competition for Ago2 [47]. For piRNA, the endo-siRNA pathway may suppress the expression of piRNAs in the ovary somatic sheet cell line of *Drosophila* [15]. In summary, the expression profiles of 25 genes of the RNAi core machinery (including genes from miRNA pathway, siRNA pathway and piRNA pathway) were screened in three experimental conditions, spatiotemporal samples, artificial synthesized dsRNA treated samples, and artificial synthesized miRNA treated samples. The expanded genes such as *Pasha*, *Dicer1*, *loquacious*, *Ago1*, *Piwi*, and *Ago3*, showed significant response to dsRNA and/or miRNA, implying a complex network of RNAi core machinery in aphids. Further studies should investigate the contribution of these expanded genes (*loquacious*, *Ago1*, *Piwi*, and *Ago3*) to the activity of the siRNA pathway. This information will help determine the potential of RNAi for aphid pest control. In addition, the siRNA pathway is reported to be involved in defense of plant viruses in aphids [48] and these genes could be responsible for immunity that may be able to against the viruses before they are transmitted to plants. This possibility will also require further studies.

## 5. Conclusions

The expressions of 25 genes of RNAi in three RNAi pathways (siRNA, miRNA, and piRNA) were detected in spatiotemporal samples, artificially synthesized dsRNA and miRNA-treated samples. The results from different development stages and different tissues indicated that the miRNA pathway and piRNA pathways are important in the regulation of insect development and reproduction. A complex network of RNAi cores genes in the three sub-pathways was found based on data from the artificially synthesized dsRNA and miRNA treated samples.

## Supplementary Materials

The following are available online at https://www.mdpi.com/2075-4450/11/2/70/s1, Table S1: Primers used for dsRNA synthesis and RT-qPCR, Table S2: Accession number of genes used in this study, Figure S1: The relative mRNA expression profiles of *Hunchback* upon ds*HB* administration, Figure S2: Relative expression profiles of *miR-3024* upon *miR-3024* agomir/antagomir administration.
