# Supplementary material for: Expression Dynamics of Core RNAi Machinery Genes in Pea Aphids Upon Exposure to Artificially Synthesized dsRNA and miRNAs
Source: Insects. 2020 Jan 21;11(2):70. doi: 10.3390/insects11020070 (PMC7074054; doi:10.3390/insects11020070)
Supplement: Supplementary file 1 [file insects-11-00070-s001.pdf]

Table S1. Primers used for dsRNA synthesis and RT-qPCR.

**Expriment**	**Primer names and sequence (5′ to 3′)**	**Amplification efficiency**
dsRNA synthesis	ds*Hunchback*-F: taatacgactcactatagggGCATCACCGTACTCGATGCAA	
	ds*Hunchback*-R: taatacgactcactatagggGGGAGCCACTGAGTCTCTGCT	
	ds*GFP*-F: taatacgactcactatagggGGTGAGCAAGGGCGAGGAGCTG	
	ds*GFP*-R: taatacgactcactatagggGGCTCGATGCGGTTCACCAG	
qPCR analysis	*EF1α*-F: CTGTGCTTATTGTCGCTGCT	98.0%
	EF1α-R: TCGCTGTATGGTGGTTCAGT	
	RPS20-F: AAGTGTGTGCTCCGAGATGA	104.0%
	RPS20-R: CAGCAATGACACCGGGTTC	
	q*Ago1a*-F: CCTGTATTTGATGGTCGCAACA	96.5%
	q*Ago1a*-R: GCGGCCTTCTAATGCTTCTT	
	q*Ago1b*-F: CGGCATCACACCAGATTGTT	103.4%
	q*Ago1b*-R: GGCCGACTAGTTCCCTGAAT	
	q*Dicer1a*-F: GGAAGAGCCTAGACCTGGAA	93.1%
	q*Dicer1a*-R: TCCCAAACCCATGAACGGAT	
	q*Dicer1b*-F: TGACAGATCTTCGTTCAGCAC	105.0%
	q*Dicer1b*-R: CGGTGCCTCAACATCTTCAG	
	q*Pasha1*-F: CACGTGGAGTTGCGGGAATA	98.7%
	q*Pasha1*-R: TCTCGACGACGATTACACCG	
	q*Pasha2*-F: GCAGAGAAACTTTGGTGAGTGT	103.3%
	q*Pasha2*-R: ACCGTAAAGCCGTAACAACG	
	q*Pasha3*-F: TGGTAAAAGCTGATCGAAGGC	104.4%
	q*Pasha3*-R: TGGCTGAGTTTTGAGTACACG	

	q*Pasha4*-F: TCGCTGCACTATGACTGTAGA	107.7%
	q*Pasha4*-R: GATGAGTTCTACATTGTTCGGC	
	q*Drosha*-F: CGATCAGTAGACGGTGGTGA	101.3%
	q*Drosha*-R: AACGCACTATTGGCAGCATT	
	q*Exportin5*-F: CCACTGTGCGTTCAATGTCA	106.0%
	q*Exportin5*-R: AGCAGAATTGACCAGGCTCA	
	q*Loquacious1*-F: TCCACCGTTAACCACAGTCT	103.1%
	q*Loquacious1*-R: GGAGATTTCAAGCTGTCCTGT	
	q*Loquacious2*-F: CGATTGGCATGCTTCAGGAA	98.1%
	q*Loquacious2*-R: TCTACATTCTCAGCCGCTCC	
	q*Ago2*-F: CAGGCACCACAGAAACAAAA	94.3%
	q*Ago2*-R: AATTTGTTGACCGCCTTGAG	
	q*Dicer2*-F: TGGTCTCAACAGCAATGGAA	97.5%
	q*Dicer2*-R: AATACGGGGACGTTCATCAG	
	q*R2D2*-F: AGAAGGACAAGCCCATGCTA	109.7%
	q*R2D2*-R: TGGATTCCCTTCCATTACCA	
	q*Ago3a*-F: TATGCCCTTGTGAGACGGAG	108.2%
	q*Ago3a*-R: CTTCTTCCGCGAGCAACTTG	
	q*Ago3b*-F: AATGATGCGTCCAATCCCAG	101.0%
	q*Ago3b*-R: GCCGTTCCTTCATCTCTTGG	
	q*Piwi1*-F: CAACGGCGGACAAGACATTC	106.9%
	q*Piwi1*-R: AGTTCTTTGTGTGCGCTCAA	
	q*Piwi2*-F: AGAGCAGTACCGTCACAAGT	102.9%
	q*Piwi2*-R: TCCGCTGGCTATCATGACAA	

	q*Piwi3*-F: CTTTGGCCCGGGTATGAAAC	104.9%
	q*Piwi3*-R: TCATCACCGTGGTACCAACA	
	q*Piwi4*-F: AGCAGGTCCCAGTGAAAGAA	102.8%
	q*Piwi4*-R: CACAAGTGAACATGGGTGCA	
	q*Piwi5*-F: GCAGTACCGTCCCAAGTAGT	106.7%
	q*Piwi5*-R: TCCGCTGGCTATCATGACAA	
	q*Piwi6*-F: GGAGCAAAGGGAAAACAGGG	107.5%
	q*Piwi6*-R: GGTGGATCAAATCGGCTTCC	
	q*Piwi7*-F: CATGACTGATTTGGCGGTGT	100.2%
	q*Piwi7*-R: GCTAGATGTCCAATCTGCTGC	
	q*Piwi8*-F: AAACAATGCAGCTACGCCAA	99.7%
	q*Piwi8*-R: CCCGAGTGACTCTGCTGTAA	
	miR-3024-F: CTTCTTTGGGATTTAATAGAGCCGG	95.57%
	U6: CGATACAGAAGATTAGCATGG	92.61%

Table 2. Accession number of genes used in this study.

Gene	Accesion number
Dicer2	XM_008189326
R2D2	NM_00162172.2
Ago2	XM_001944817.3
Drosha	XM_003247865
Pasha1	XM_016808185.3
Pasha2	XM_001947368.8
Pasha3	XM_001948642.4
Pasha4	XM_001951974.4
Exp-5	XM_003241026.1
Dicer1a	XM_001944314.1
Dicer1b	XM_016807644.1
Loquacious1	XM_016804908
Loquacious2	XM_016802268
Ago1a	XM_003240572.3
Ago1b	XM_001944279.4
Piwi1	XM_003243306
Piwi2	XM_016805294
Piwi3	XM_008184469
Piwi4	XM_01680282833
Piwi5	XM_016804870
Piwi6	XM_008188500
Piwi7	XM_001948406
Piwi8	XM_001947555.4
Ago3a	XM_016800535
Ago3b	XM_003245551
EF1α	XM_001948705.4
RPS2	NM_001162819.2
U6	KX638479
Hunchback	NM_001162510.1

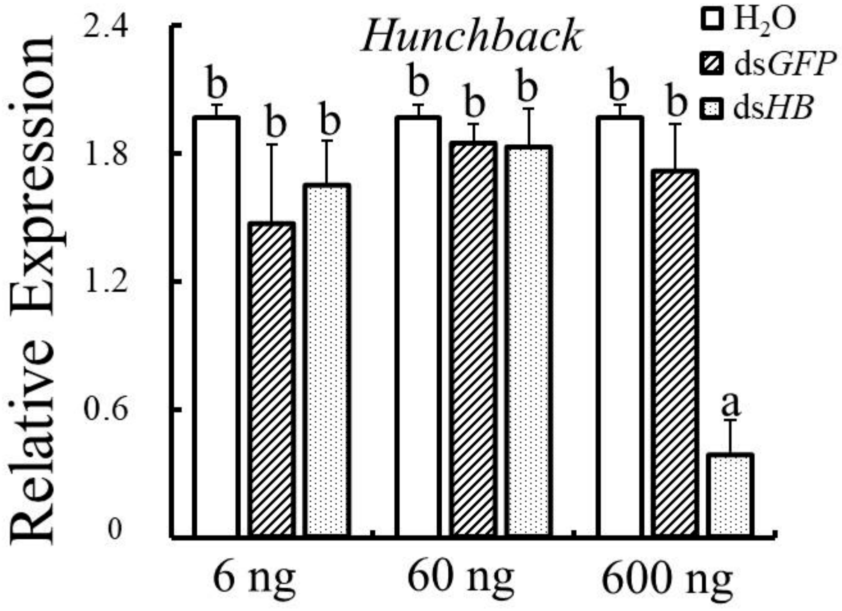

Figure S1. The relative mRNA expression profiles of *Hunchback* upon ds*HB* administration. The mean (±SE) expression level is based on four biological replicates. The relative expression was calculated based on the value of the reference genes. Lowercase letters above each bar indicate significant differences (one-way ANOVA followed by Tukey's honestly significant difference multiple comparison test; P < 0.01).

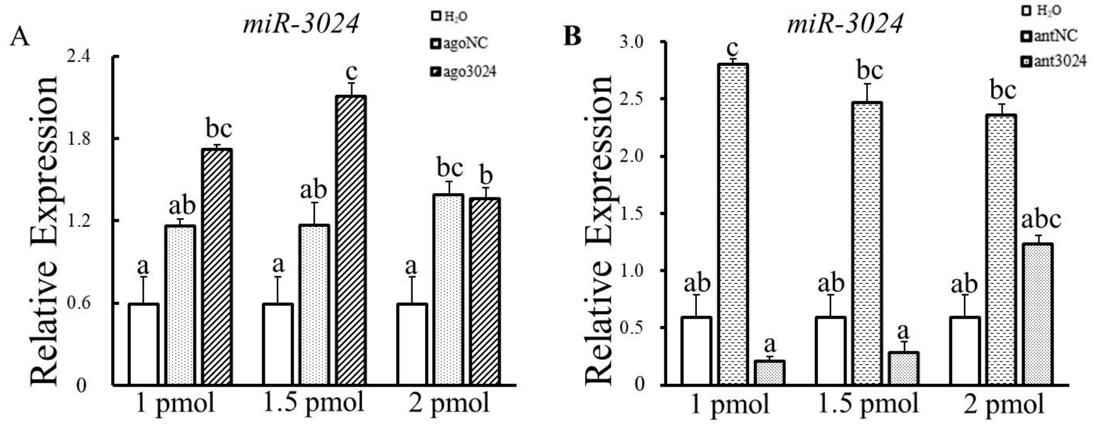

Figure S2. Relative expression profiles of *miR-3024* upon *miR-3024* agomir (A) and antagomir (B) administration. The mean (±SE) expression level is based on four biological replicates. The relative expression was calculated based on the value of the reference genes. Lowercase letters above each bar indicate significant differences (one-way ANOVA followed by Tukey's honestly significant difference multiple comparison test; P < 0.01).
